# Three-dimensional magnetic resonance imaging-based statistical shape analysis and machine learning-based prediction of patellofemoral instability

**DOI:** 10.1038/s41598-024-62143-7

**Published:** 2024-05-18

**Authors:** Keita Nagawa, Kaiji Inoue, Yuki Hara, Hirokazu Shimizu, Saki Tsuchihashi, Koichiro Matsuura, Eito Kozawa, Naoki Sugita, Mamoru Niitsu

**Affiliations:** 1 Department of Radiology, Saitama Medical University, 38 Morohongou, Moroyama-Machi, Iruma-Gun, Saitama, Japan; 2 Department of Orthopedics, Saitama Medical University, 38 Morohongou, Moroyama-Machi, Iruma-Gun, Saitama, Japan

**Keywords:** Patellofemoral instability, Statistical shape analysis, Three-dimensional magnetic resonance image, Generalized Procrustes analysis, Medical research, Trauma

## Abstract

This study performed three-dimensional (3D) magnetic resonance imaging (MRI)-based statistical shape analysis (SSA) by comparing patellofemoral instability (PFI) and normal femur models, and developed a machine learning (ML)-based prediction model. Twenty (19 patients) and 31 MRI scans (30 patients) of femurs with PFI and normal femurs, respectively, were used. Bone and cartilage segmentation of the distal femurs was performed and subsequently converted into 3D reconstructed models. The pointwise distance map showed anterior elevation of the trochlea, particularly at the central floor of the proximal trochlea, in the PFI models compared with the normal models. Principal component analysis examined shape variations in the PFI group, and several principal components exhibited shape variations in the trochlear floor and intercondylar width. Multivariate analysis showed that these shape components were significantly correlated with the PFI/non-PFI distinction after adjusting for age and sex. Our ML-based prediction model for PFI achieved a strong predictive performance with an accuracy of 0.909 ± 0.015, and an area under the curve of 0.939 ± 0.009 when using a support vector machine with a linear kernel. This study demonstrated that 3D MRI-based SSA can realistically visualize statistical results on surface models and may facilitate the understanding of complex shape features.

## Introduction

Patellofemoral instability (PFI) is a pathological condition characterized by recurrent patellar subluxation or dislocation. It mainly occurs in young and active populations, and accounts for approximately 3% of all knee injuries^1,2^. PFI is a multifactorial disease. The known risk factors are trochlear dysplasia, patella alta, increased tibial tubercle-to-trochlear groove distance, abnormal patellar lateral tilt, and coronal and torsional malalignment^3–6^. The risk of recurrent instability varies widely after primary dislocation, ranging from 11 to 60%^7–10^. Various nonsurgical and surgical treatments are available to treat these underlying causes.

The imaging modalities for the study of knee conditions, including PFI, are plain radiographs, computed tomography (CT), and magnetic resonance imaging (MRI)^6,11,12^. Plain radiographs are essential for visualizing bones and surveying their abnormalities at a low radiation dose and cost. As for PFI, several radiographic signs have been reported, including the crossing sign, trochlear bump or depth, and patella alta index^5,27^. However, plain radiographs are two-dimensional and may not adequately visualize complex articular structures. Sequential imaging modalities such as CT and MRI are preferred because they allow for more precise and more detailed visualization of the joint tissues. CT is preferred for investigating bones, whereas MRI is mainly used for soft tissues. However, MRI may also play an alternative role^11^. For example, MRI can detect several bone abnormalities, such as contusions, fragments, deformities, and surrounding tissue abnormalities. The importance of MRI has increased with the development of high-resolution functional imaging techniques. In knee studies, our institution routinely uses a three-dimensional (3D) T2 star-weighted fast-field echo (T2* FFE) MRI sequence. Although this sequence is primarily intended for the high-resolution evaluation of cartilage, other tissues, including the bone, are also clearly visualized. The 3D T2* FFE method provides volumetric data for 3D segmentation and shape model analysis.

The development of high-resolution 3D imaging techniques has enabled researchers to characterize the morphological variations in complex biological structures using methods such as statistical shape analysis (SSA)^13–17^. A common approach when performing 3D SSA is to first acquire high-resolution images and then obtain the anatomical landmark information. These anatomical landmarks are then used in multivariate shape analyses. The general method of collecting landmark datasets involves manual registration by experts. Manual annotation of landmarks is laborious, time-consuming, low-throughput, and subject to inter-observer biases^18^. Although automated approaches have been considered, most depend on high-end hardware and specialized MATLAB software and require more time to produce results than manual landmark annotations^18,19^. Considering these points, we propose a cutting-edge open-source software to analyze 3D morphological data, SlicerMorph, on the 3D Slicer platform^20^. This application contains a set of modules for automatic registration and SSA, among which a tool called automated marking through point-cloud alignment and correspondence analysis (ALPACA) enables fast and accurate automated landmarking through point-cloud-based deformable model registration^21^.

The 3D SSA of knees with PFI has been previously studied. Some studies used CT^22,23^, whereas others used MRI^24–26^. Van Haver et al. conducted a CT-based study of trochlear dysplasia^22^. In their study, the mean shape models of normal and trochlear dysplastic femur bones were obtained using generalized Procrustes analysis (GPA) and principal component analysis (PCA). Subsequently, the researchers evaluated the difference between the two mean models and found that the trochlea was anteriorized, proximalized, and lateralized, and that the mediolateral width and notch width were decreased in the trochlear dysplastic femur compared to the normal femur. They also developed an automated classification of trochlear dysplastic and normal femurs using a combination of principal components, which achieved a sensitivity of 85% and specificity of 95%. MRI-based SSA of PFI has also been reported, and similar results have been obtained. Fitzpatrick et al. created an MRI-based 3D shape model of the patellofemoral joint and characterized shape variations in the patella alta-baja and depth of the sulcus groove^26^. Considering the inevitable exposure to radiation in CT scans, and the versatility and utility of MRI studies, MRI-derived SSA is favorable. Furthermore, MRI can visualize cartilage as well as bone; therefore, 3D shape models of bone and cartilage can be built using MRI-based SSA.

In this study, we aimed to build 3D MRI-based bone and cartilage models of normal femurs and femurs with PFI, and perform SSA to compare the two models using the SlicerMorph package. We also conducted a multivariate analysis of shape components to identify the independent shape characteristics associated with PFI, evaluated confounding factors such as age and sex, and performed an adjusted multivariate analysis. Moreover, we developed a machine learning (ML)-based prediction model for PFI using shape components derived from the SSA.

## Methods

### Subjects and image acquisitions

This study was approved by the Research Ethics Committee of Saitama Medical University Hospital (approval number 2023–047). All experiments were performed in accordance with relevant guidelines and regulations. The requirement for informed consent was waived by the Research Ethics Committee of Saitama Medical University Hospital.

After receiving institutional review board approval, we identified a consecutive series of patients under 40 years of age who underwent non-contrast non-arthrogram knee MRI between January 2017 and September 2021, with an order from the Department of Orthopedics in our hospital. All patients diagnosed with PFI at our institution were included in this study. Although all patients initially underwent MRI for suspected PFI-related injury, those with knee pain due to other etiologies such as fracture, arthritis, chondrosis, osseous stress response, or high-grade ligament sprains were excluded. A group of age- and sex-matched controls were also identified, defined as those with normal knee MRI findings and symptoms that did not involve the patellofemoral compartment of the knee. Controls with fractures, arthritis, chondrosis, osseous stress response, or high-grade ligament sprains were excluded.

All MRI scans were performed using a 3.0-T system (Ingenia Elition, Philips Healthcare, The Netherlands) with a vendor-specific 16-channel knee coil. In addition to the routine knee protocol including axial, sagittal, and coronal proton density sequences, a 3D T2* FFE sequence was performed in all patients. The specific implementation protocol for 3D T2* FFE was as follows: repetition time, 15 ms; echo time, 5 ms; flip angle, 30°; slice thickness, 1.5 mm; field of view, 15.0 × 17.1 cm.

### Construction of the 3D surface models

To create smooth 3D surface models of the knee bone and cartilage, MR images were loaded into an open-source software (ITK-SNAP version 3.8.0) in the Digital Imaging and Communications in Medicine (DICOM) format. Subsequently, the areas of femoral bone and cartilage were manually delineated in each slice. Two radiologists with 7 and 6 respective years of experience performed this independently, and subsequently reached a consensus. Both radiologists were blinded to the clinical information. From the delineated contours, a 3D surface model of each bone and cartilage was reconstructed and saved as a standard 3D model in the Neuroimaging Informatics Technology Initiative (NIfTI) file format (*.nii.gz). These 3D surface models were subsequently loaded into another open-source software package (3D Slicer version 5.0.3) for 3D landmark-based shape model analysis.

### Application of the automated 3D landmarking and SSA

To develop an automated 3D landmark-based SSA, we performed the following procedures: (a) creation of a reference landmark set (automatic landmark placement on the surface model of the normal femur bone) using the pseudo-landmark generator algorithm, (b) automatic landmark registration using the ALPACA algorithm, (c) evaluation of shape differences based on GPA and PCA, (d) multivariate analysis of shape components, and (e) development of an ML-based prediction model for PFI.

#### Step 1. Creation of a reference landmark dataset

We started by obtaining reference landmark data using a pseudo-landmark generator module.

When developing automated landmarking methods, it is common to use a dataset of manually digitized samples as the reference set. However, we believe that performing automatic landmarking on a reference model is beneficial. The pseudo-landmark generator module generated a set of landmarks at regular intervals on the external surface of the sample model. We then applied this module to a shape model of a normal femur bone and obtained a reference sample of the landmarks.

#### Step 2. Automatic landmark registration using ALPACA algorithm

We applied the ALPACA framework to the surface bone models of the normal and PFI cases and obtained the corresponding landmark datasets. More specifically, point-cloud-based alignment and registration proceeded in the following steps: (a) a source model (the reference model used in Step (1) and a target model were down-sampled into corresponding dense point clouds that were then rigidly aligned with each other; (b) a deformable transformation was subsequently performed, in which the source model was deformed to match the target model; and (c) the placement of landmarks in the source model was projected onto the target model using the point correspondence method. By repeating this registration step for all models of the normal and PFI cases, landmark datasets were obtained for subsequent analysis.

#### Step 3. Evaluation of shape differences based on GPA and PCA

To evaluate the shape differences between the normal and PFI cases, we implemented a GPA-based transformation of the surface model and landmark coordinate system and we calculated and compared the mean shape of each femur model. Pointwise signed distances between the mean normal and PFI surface models were calculated and projected onto the surface of the mean normal femur model. PCA was applied to the GPA-aligned coordinate system to explain the shape variations within each group, and the shape characteristics of each principal component were evaluated. The GPA and PCA module on SlicerMorph visualized the 3D shape variations of each principal component, and we subsequently plotted the shape corresponding to mean ± 2.5 standard deviations (SD).

#### Step 4. Multivariate analysis of shape components

In further studies, we conducted a multivariate analysis of the shape components. The main shape components obtained for a joint dataset of normal femurs and femurs with PFI in Step 3 were subsequently loaded into the Python platform along with the corresponding standard scores and parameters such as age, sex, and PFI/non-PFI. Multivariate analysis was performed to identify the components that had a strong relationship with PFI. In addition, we used multivariate analysis to evaluate the relationship between the shape components and confounding variables such as age and sex. To identify components that had an independent relationship with PFI, we also conducted a multivariate analysis adjusting for age and sex.

#### Step 5. ML-based prediction model for PFI

Following the results of Step 4, we determined which significant shape components exhibited a strong relationship with PFI, as well as which principal components were successful in discriminating between PFI and non-PFI femurs. A classification model was developed using these important shape components and four representative ML classifiers: linear discriminant analysis (LDA), support vector machine (SVM), k-nearest neighborhood (k-NN), and random forest (RF). In the SVM algorithm, various kernel functions provide different decision-making abilities and versatility. In this study, we adopted two representative kernels – linear and rbf – separately and compared their results. Thus, we tested five different ML classifiers: LDA, SVM with linear and rbf kernels, k-NN, and RF. The number of shape components was reduced to five to prevent overfitting due to the small sample size used in our study. The performance of the classifiers was validated using a five-fold cross-validation method and evaluated using receiver operating characteristic (ROC) analysis and the area under the curve (AUC). Accuracy, sensitivity, and specificity were calculated based on the confusion matrix of the classification results.

All procedures were performed using the SlicerMorph module of the 3D Slicer platform. Multivariate analyses and ML-based predictions were performed using an open-source software package (Python scikit-learn 0.22.1). Statistical significance was set at P < 0.05. For the implementation details, we referred to methods described in previous studies^20–22^.

## Results

Twenty MRI scans of femurs with PFI obtained from 19 patients (sex ratio, 8/12 [male/female]; age, 20.5 ± 6.8 [mean ± SD]; side ratio, 7/13 [right/left], including both sides of the femur in one patient) and 31 MRI scans of normal femurs obtained from 30 patients (sex ratio, 13/18; age, 20.5 ± 3.9; side ratio, 16/15, including both sides of the femur in one patient) were used in the study. All the participants were Japanese.

### Evaluation of shape differences based on GPA and PCA

**Figure 1:**
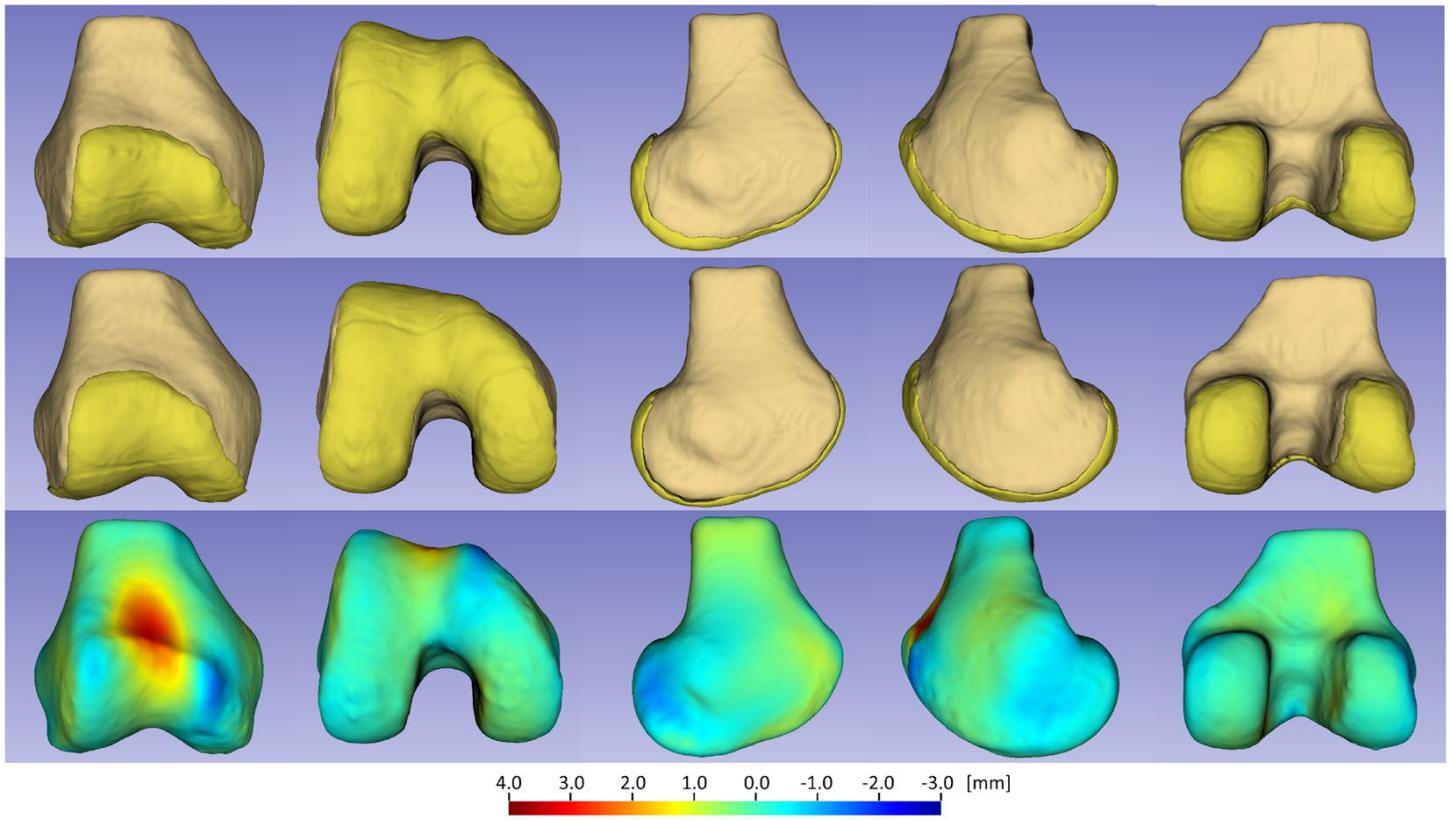
Mean shape models of the normal distal femur (upper row) and the distal femur with patellofemoral instability (middle row) as well as a pointwise distance map between the two models (lower row) are shown. From left to right, the 3D shape models are exhibited in the following order: front, bottom, lateral, medial and rear side.

The mean normal and PFI surface models as well as the pointwise signed distances between them are shown in Fig. 1. In the anterior part of the distal femur, the trochlea was elevated anteriorly, proximalized, and lateralized in the PFI group compared with the normal group. Trochlear anteriorization in PFI was mainly observed in the middle of the trochlear floor (Fig. 1). Proximalization and anteriorization were highlighted by the deep red-colored area (showing significant positive values) at the central floor of the trochlea, whereas lateralization was demonstrated by the slightly yellow area (showing slightly positive values) on the lateral side of the trochlea and the blue area (showing negative values) on the medial side of the trochlea. The intercondylar notch was narrower in the PFI group than in the normal group, with a slightly yellow area on the notch side and a blue area on the opposite side of both condyles. Almost all the other areas of the distal femur were green, indicating that almost no pointwise distances were observed between the PFI and normal femoral models.

**Figure 2:**
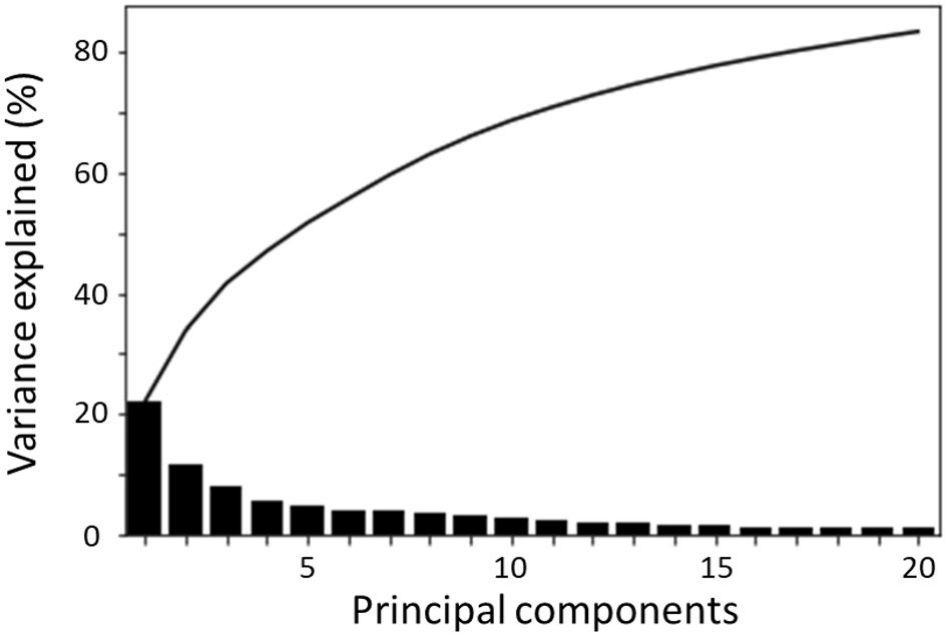
Cumulative variance (line plot) and fraction of variance (bar plot) explained by principal components of the statistical shape models of the distal femur with patellofemoral instability.

**Figure 3:**
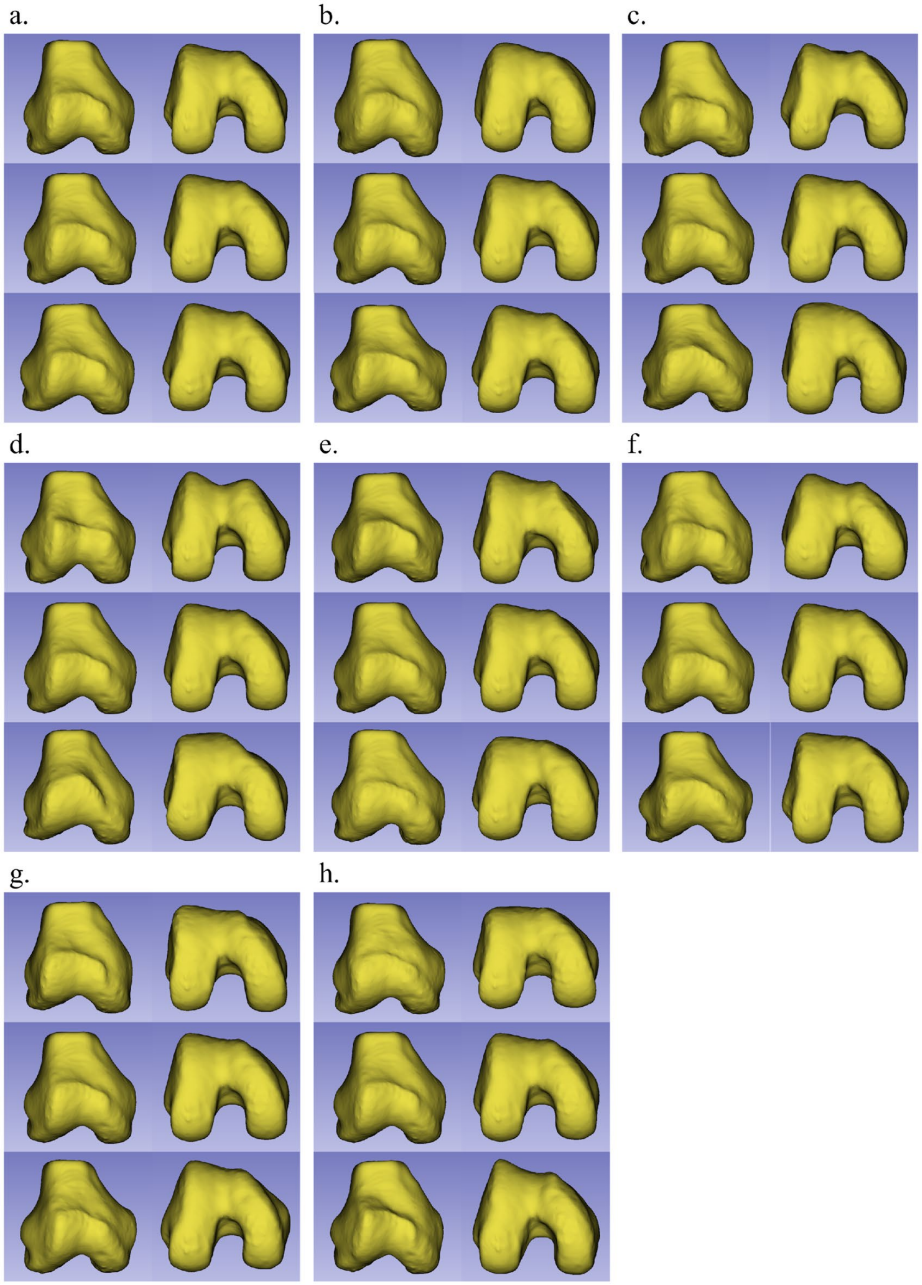
The shape variations (corresponding to mean ± 2.5 standard deviation [SD]) from the three first principal components (the first (**a**), second (**b**), third (**c**), fifth (**d**), sixth (**e**), eighth (**f**), ninth (**g**) and tenth (**h**) components) of the models of the distal femur with patellofemoral instability are shown. For each component, the front and bottom sides of the models are exhibited, in order of + 2.5 SD, mean, and –2.5 SD, from top to bottom.

The results of the PCA of the distal femur of the PFI group with the GPA-aligned coordinate system are shown in Figs. 2 and 3. The cumulative variance and the variance for each mode are shown in Fig. 2. The first three and ten principal components explained 41.6% and 68.7% of the observed population variance, respectively.

In Fig. 3, the 3D shape variations (corresponding to mean ± 2.5 SD) of the first three principal components are shown. The first principal component, which accounted for 22.0% of the variation, concerned the medial and lateral epicondyles and defined the horizontal size of the distal femur rather than the trochlea and condyles. The second and third principal components, accounting for 11.8 and 7.8% of the variation, respectively, were related to shape variations in the sulcus angle and intercondylar width. In the second component, the convexity of the anterior trochlea was evident in the + 2.5 SD shape, whereas a concave anterior trochlea and large intercondylar notch width were observed in the – 2.5 SD shape. In the third component, smaller condyles and a broad intercondylar notch were observed in the + 2.5 SD shape, whereas a convex anterior trochlea, larger condyles, and a narrow intercondylar notch were observed in the − 2.5 SD shape. In both the fifth and eighth components, a narrow intercondylar notch and concavity of the anterior trochlea were observed in the + 2.5 SD shape, whereas the opposite was observed in the – 2.5 SD shape. These shape changes were more evident in the fifth component than in the eighth component. The tenth component was mainly associated with the anterior trochlea, which had a flattened morphology in the + 2.5 SD shape, but was anteriorized, especially in the medial part of the trochlear floor, in the − 2.5 SD shape.

### Multivariate analysis of shape components

The results of the multivariate analysis of the shape components are summarized in Table 1. The second, third, fifth, eighth, and tenth principal components were significantly correlated with the distinction between PFI and non-PFI cases. These components showed significant correlations with the PFI/non-PFI distinction, even after adjusting for age and sex.

**Table 1:** Summary of the multivariate analysis of shape components for PFI.

			Adjusted by age and sex	
PC	RC	P value	RC	P value
PC 1	− 0.18	0.914	0.41	0.807
PC 2	6.98	0.004	11.90	0.001
PC 3	− 9.81	0.001	− 8.07	0.007
PC 4	2.08	0.542	3.17	0.347
PC 5	− 23.17	< 0.001	− 22.31	< 0.001
PC 6	− 8.95	0.029	− 6.52	0.117
PC 7	4.45	0.271	3.25	0.399
PC 8	− 16.35	< 0.001	− 21.30	0.001
PC 9	2.20	0.630	7.34	0.140
PC 10	18.93	< 0.001	12.54	0.022

*PC* principal component, *PFI* patellofemoral instability, *RC* regression coefficient.

We also examined the association with age and sex (Table 2). No significant correlation between shape factors and age was observed. Regarding sex, the second, eighth, and ninth principal components were correlated with sex after adjusting for PFI and age.

**Table 2:** Summary of the multivariate analysis of shape components for age and sex.

	Age				Sex			
			Adjusted by sex and PFI				Adjusted by age and PFI	
PC	RC	P value	RC	P value	RC	P value	RC	P value
PC 1	22.50	0.627	12.53	0.796	2.70	0.093	2.48	0.113
PC 2	− 60.57	0.341	− 111.07	0.233	13.65	< 0.001	16.10	< 0.001
PC 3	-5.39	0.945	− 25.41	0.758	5.41	0.046	2.61	0.385
PC 4	52.51	0.576	32.63	0.739	5.38	0.099	5.60	0.080
PC 5	152.05	0.133	121.63	0.264	8.22	0.02	0.44	0.932
PC 6	51.24	0.639	15.60	0.896	9.64	0.013	6.68	0.095
PC 7	31.02	0.778	41.28	0.712	-2.77	0.462	− 1.71	0.646
PC 8	− 207.14	0.081	− 120.86	0.461	-23.32	< 0.001	− 26.57	< 0.001
PC 9	− 103.01	0.413	− 150.55	0.289	12.85	0.004	14.22	0.002
PC 10	259.01	0.058	300.69	0.054	− 11.27	0.017	− 7.64	0.156

*PC* principal component, *PFI* patellofemoral instability, *RC* regression coefficient.

### ML-based prediction model for PFI

**Figure 4:**
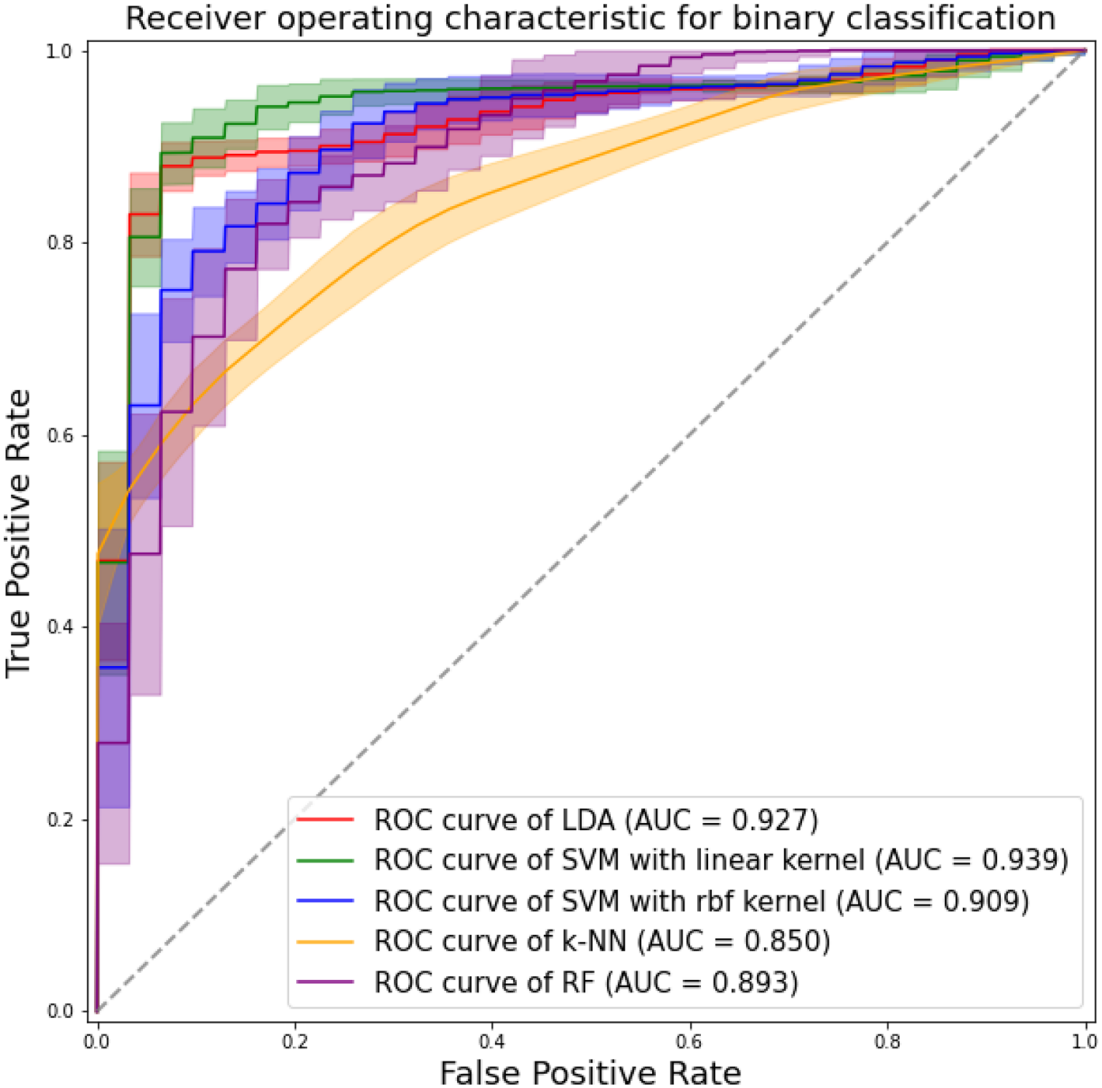
The receiver operating characteristic (ROC) curves and area under the curve (AUC) values of machine learning-based classification models for differentiating between patellofemoral instability (PFI) and non-PFI groups using shape components derived from three-dimensional statistical shape analysis, with a linear discriminant analysis (LDA) classifier (red line), support vector machine (SVM) classifier with a linear kernel (green line) and rbf kernel (blue line), k-nearest neighborhood (k-NN) classifier (orange line), and random forest (RF) classifier (purple line).

The results of the ML-based prediction attempts are summarized in Table 3, and the ROC curves are shown in Fig. 4. Among the five ML classifiers we studied, the most favorable performance was observed in the SVM classifier with a linear kernel, with an accuracy of 0.909 ± 0.015 and an AUC of 0.939 ± 0.009. The confusion matrices of the prediction attempts are summarized in Supplementary Fig. S1.

**Table 3:** Results of the machine learning-based prediction model for PFI.

	Accuracy	Sensitivity	Specificity	AUC
LDA	0.893 ± 0.018	0.827 ± 0.033	0.950 ± 0.018	0.927 ± 0.009
SVM with linear kernel	0.909 ± 0.015	0.884 ± 0.030	0.931 ± 0.012	0.939 ± 0.009
SVM with rbf kernel	0.845 ± 0.022	0.776 ± 0.032	0.905 ± 0.030	0.909 ± 0.013
k-NN	0.778 ± 0.019	0.656 ± 0.032	0.884 ± 0.027	0.850 ± 0.017
RF	0.823 ± 0.023	0.799 ± 0.034	0.844 ± 0.029	0.893 ± 0.018

*AUC* area under the curve, *k-NN* k-nearest neighborhood, *LDA* linear discriminant analysis, *PF* patellofemoral instability, *RF* random forest, SVM support vector machine.

## Discussion

In this study, we created 3D MRI-based shape models of femurs with PFI and normal femurs and compared the two models using SSA tools. Elevation of the trochlea in the PFI models compared with the normal models was observed on the central floor of the proximal trochlea. Furthermore, we applied PCA to the GPA-aligned coordinate system to evaluate the shape variations in the PFI group and found that several principal components were related to shape variations in the trochlear floor and intercondylar width. We used a multivariate analysis to show that these shape components were significantly correlated with the PFI/non-PFI distinction after adjusting for age and sex. In addition, we developed an ML-based prediction model for PFI using these shape components and obtained a favorable predictive performance.

The morphological analysis of PFI has been previously reported. Earlier studies examined radiographic signs and several specific measurements, such as the crossing sign, trochlear bump or depth, and patella alta index^5,27^. However, these morphological parameters are primarily qualitative or quasi-quantitative, leading to high inter-reader variability and insufficient reliability. To overcome these shortcomings and understand complex articular structures more realistically, computer-aided morphological analyses using 3D shape models have been conducted^28,29^. Using 3D models of bone and articular cartilage, Yamada et al. demonstrated the proximalization and lateralization of the trochlear cartilage and the associated wider convex trochlea in PFI^29^. However, the approach used in this study was still based on a limited set of discrete geometric variables such as angles, heights, and distances. SSA is thought to be suitable for visualizing and systematically understanding anatomical structures and their changes^13^. SSA can be used to estimate shape variability within samples, acquire mean shapes from groups, and perform clustering and testing for differences between groups. Landmark-based techniques, also called point distribution models, are often used to identify anatomical characteristics and align these point cloud sets using Procrustes transformations and statistics such as PCA to estimate shape variations. An early two-dimensional study analyzed the curvature of the trochlear groove in PFI ^30^. In a more recent study, Van Haver et al. reported a CT-based 3D SSA to obtain mean shape models of normal and trochlear dysplastic femurs and evaluated their differences^22^. Although this 3D SSA of PFI was mostly based on CT, some studies have used 3D MRI. Fitzpatrick et al. created an MRI-based 3D shape model of the patellofemoral joint and showed that the principal components concerned variations in the patellar position and depth of the sulcus groove^26^. In a more recent study, Yang et al. performed an MRI-based 3D SSA and described a shallower trochlear groove and decreased anteroposterior and mediolateral dimensions of the femoral condyles in femurs with PFI^25^.

The largest risk factor for PFI is trochlear dysplasia, and the proximal trochlea has been shown to be more anterior in femurs with PFI than in normal femurs^27^. Van Harver et al. performed a 3D SSA on trochlear dysplasia and demonstrated that the largest differences between the mean normal and trochlear dysplastic femur models were observed in the proximal part of the trochlea^22^. This study suggested that the proximal trochlea in cases of trochlear dysplasia was anteriorly elevated compared with the normal trochlea. Furthermore, trochlear anteriorization was most pronounced on the central floor of the proximal trochlea^22^. In our study, similar to these findings, trochlear anteriorization in PFI was mainly observed in the middle of the trochlear floor. We showed that anteriorization gradually decreased toward the notch, which is consistent with the findings of a previous study. Lateralization of the trochlea has also been reported, in addition to anteriorization and proximalization. Van Harver et al. also demonstrated a lateral shift of the trochlea in a trochlear dysplasia model, although this was less obvious than anteriorization and proximalization^22^. In the present study, trochlear lateralization was suggested by a slight lateral shift in the medial and lateral sides of the trochlea. It has been suggested that the articular cartilage of the trochlea may adapt to contact the articular cartilage of the patella^29^. Therefore, anteriorization, proximalization, and lateralization of the trochlea may be associated with a high-riding and lateralized patella. However, in the present study, we could not perform correlation tests for patellar position.

In this study, we performed PCA to assess the shape variations in the PFI group. Our findings showed that the first principal component concerned the medial and lateral epicondyles, defining the horizontal size of the distal femur rather than the trochlea or condyle. The first component is usually the size variation, which is not necessarily related to PFI, as described in previous studies^22,26^. Our study showed that the second and third principal components were related to the shape variations in the sulcus angle and intercondylar width, which were related to the actual shape changes of PFI. Similar results were obtained in a previous study which demonstrated that the second and third components were related to the sulcus angle and intercondylar width, respectively ^22^. It has been reported that there is a close relationship between the sulcus angle and intercondylar notch width^27^, and both are related to the anteriorization of the central trochlea, which is the main factor in trochlear dysplasia, as described above.

In this study, we performed a multivariate analysis to examine which shape features were correlated with the PFI/non-PFI distinction. The second and third principal components described above were significantly correlated with the distinction between PFI and non-PFI cases. In addition, other shape components, such as the fifth, eighth, and tenth components, were also correlated with the PFI/non-PFI distinction. These shape features also describe variations in the depth of the anterior trochlear floor and the intercondylar notch width. Multivariate analyses of shape variance can be useful when comparing groups with several confounding factors, as is the case with ordinary statistical analyses. In the current study, we chose age and sex as possible confounding parameters other than PFI/non-PFI. We confirmed that the principal components that significantly contributed to the PFI/non-PFI distinction (i.e., the second, third, fifth, eighth, and tenth components) were independent discriminatory factors even after adjusting for age and sex. We further examined age- and sex-related shape components using multivariate analysis. No significant correlation between shape factors and age was observed in this study. A previous study showed that prior to the progression of osteoarthritis, knee shape does not change significantly over time ^31^. Although age-related changes may inevitably include changes associated with osteoarthritis, the current study did not include people over 50 years of age, in whom osteoarthritis often occurs; therefore, there is no need to consider this factor. Regarding sex, several shape components showed correlations in this study; the second, eighth, and ninth principal components were significantly correlated with sex after adjusting for PFI and age. There have been several reports on sex-related changes in knee shape, which showed that compared with males, females had deepening of the intercondylar fossa, broader shaft width relative to epicondylar width, and decreased inferior protrusion of the medial and lateral condylar heads relative to the patellar groove^31,32^. In our study, the second and eighth principal components had overlapping correlations not only with PFI but also with sex; however, they appeared to have clearer variations in epicondylar width than the other components. Furthermore, the ninth principal component was only correlated with sex, and its shape variation was mainly related to the shaft width relative to the epicondylar width, which was consistent with previous studies on sex-related knee shape changes^31,32^. The inferior prominence of both condyles and the deepening of the intercondylar fossa were not conspicuous in our results.

In this study, we evaluated the ML-based prediction of PFI using discriminative shape features obtained from the preceding 3D SSA. Previous studies have tested predictive models using discriminant analysis for the PFI/non-PFI classification. Van Harver et al. reported an automated classification of trochlear dysplastic and normal cases using 3D SSA-based shape components, with a sensitivity of 85% and specificity of 95%^22^. Their classification model used only a linear discriminant model^22^. To our knowledge, ML classifiers other than LDA have not been previously examined. SVM exhibits high generalizability because we can select linear or non-linear kernels, and a linear kernel could be the most suited to our models. LDA also afforded a favorable outcome, but other non-linear classifiers, such as SVM with an rbf kernel, k-NN, and RF, were slightly inferior to LDA and SVM with a linear kernel. Possibly because of its small scale, this study could not build an efficient non-linear model. Furthermore, we used default hyperparameters in the non-linear classifier without parameter tuning during training. This may have led to favorable results for the linear classifier over the non-linear classifier.

The present study has several limitations. The sample size of this study was small; therefore, a sufficient statistical evaluation could not be performed. For accurate comparison, a control group of healthy volunteers matched for age, sex, and ethnic background would have been preferable. Furthermore, only the distal femur was evaluated, as the patella and proximal tibia could not be evaluated. Ideally, the overall knee shape should be considered. These issues should be addressed in future studies.

In conclusion, this study reports a 3D MRI-based SSA of PFI shape models and normal femurs. The pointwise distance map showed that the elevation of the trochlea in the PFI models compared with the normal ones was observed at the central floor of the proximal trochlea. In the PCA examining the shape variations in the PFI group, several principal components exhibited shape variations in the trochlear floor and intercondylar width. Using a multivariate analysis, we showed that these shape components were significantly correlated with the PFI/non-PFI distinction after adjusting for age and sex. We further developed an ML-based prediction model for PFI using these shape components and obtained a favorable predictive performance. 3D MRI-based SSA can provide realistic visualization of statistical results on surface models and may facilitate the understanding of complex shape features. Further studies are needed to confirm the feasibility of 3D SSA and elucidate the disease mechanism of PFI.

### Supplementary Information

Supplementary Figure S1.

## Data Availability

The datasets used and/or analyzed during the current study available from the corresponding author on reasonable request.

## References

[1] Harilainen A, Myllynen P, Antila H, Seitsalo S (1988). The significance of arthroscopy and examination under anaesthesia in the diagnosis of fresh injury haemarthrosis of the knee joint. Injury.

[2] Stefancin JJ, Parker RD (2007). First-time traumatic patellar dislocation: A systematic review. Clin. Orthop. Relat. Res..

[3] Balcarek P (2010). Anatomy of lateral patellar instability: trochlear dysplasia and tibial tubercle-trochlear groove distance is more pronounced in women who dislocate the patella. Am. J. Sports Med..

[4] Carlson VR, Sheehan FT, Shen A, Yao L, Jackson JN, Boden BP (2017). The relationship of static tibial tubercle-trochlear groove measurement and dynamic patellar tracking. Am. J. Sports Med..

[5] Diederichs G, Issever AS, Scheffler S (2010). MR imaging of patellar instability: Injury patterns and assessment of risk factors. Radiographics.

[6] Dietrich TJ, Fucentese SF, Pfirrmann CW (2016). Imaging of individual anatomical risk factors for patellar instability. Semin. Musculoskelet. Radiol..

[7] Lind M, Enderlein D, Nielsen T, Christiansen SE, Faunø P (2016). Clinical outcome after reconstruction of the medial patellofemoral ligament in paediatric patients with recurrent patella instability. Knee Surg. Sports Traumatol. Arthrosc..

[8] Nelitz M, Dreyhaupt J, Reichel H, Woelfle J, Lippacher S (2013). Anatomic reconstruction of the medial patellofemoral ligament in children and adolescents with open growth plates: surgical technique and clinical outcome. Am. J. Sports Med..

[9] Palmu S, Kallio PE, Donell ST, Helenius I, Nietosvaara Y (2008). Acute patellar dislocation in children and adolescents: A randomized clinical trial. J. Bone Joint Surg. Am..

[10] Sanders TL, Pareek A, Hewett TE, Stuart MJ, Dahm DL, Krych AJ (2018). High rate of recurrent patellar dislocation in skeletally immature patients: A long-term population-based study. Knee Surg. Sports Traumatol. Arthrosc..

[11] Figueroa D (2014). Usefulness of magnetic resonance imaging in the evaluation of patellar malalignment. Rev. Esp. Cir. Ortop. Traumatol..

[12] Ridley TJ, Hinckel BB, Kruckberg BM, Agel J, Arendt EA (2016). Anatomical patella instability risk factors on MRI show sensitivity without specificity in patients with patellofemoral instability: A systematic review. JISAKOS.

[13] Ambellan F, Lamecker H, von Tycowicz C, Zachow S (2019). Statistical shape models: Understanding and mastering variation in anatomy. Adv. Exp. Med. Biol..

[14] Dai H, Tao Y, He X, Lin H (2021). IsoExplorer: An isosurface-driven framework for 3D shape analysis of biomedical volume data. J. Vis. (Tokyo).

[15] Diamond KM, Rolfe SM, Kwon RY, Maga AM (2022). Computational anatomy and geometric shape analysis enables analysis of complex craniofacial phenotypes in zebrafish. Biol. Open.

[16] Heutinck P (2021). Statistical shape modelling for the analysis of head shape variations. J. Craniomaxillofac. Surg..

[17] Nauwelaers N (2021). Exploring palatal and dental shape variation with 3D shape analysis and geometric deep learning. Orthod. Craniofac. Res..

[18] Percival CJ (2019). The effect of automated landmark identification on morphometric analyses. J. Anat..

[19] Devine J (2020). A registration and deep learning approach to automated landmark detection for geometric morphometrics. Evol. Biol..

[20] Rolfe S (2021). SlicerMorph: an open and extensible platform to retrieve, visualize and analyse 3D morphology. Methods Ecol. Evol..

[21] Porto A, Rolfe S, Maga AM (2021). ALPACA: a fast and accurate computer vision approach for automated landmarking of three-dimensional biological structures. Methods Ecol. Evol..

[22] van Haver A (2014). A statistical shape model of trochlear dysplasia of the knee. Knee.

[23] Cerveri P, Belfatto A, Manzotti A (2020). Predicting knee joint instability using a tibio-femoral statistical shape model. Front. Bioeng. Biotechnol.

[24] Clouthier AL, Smith CR, Vignos MF, Thelen DG, Deluzio KJ, Rainbow MJ (2019). The effect of articular geometry features identified using statistical shape modelling on knee biomechanics. Med. Eng. Phys..

[25] Yang M (2020). 3D patella and femur bone shape modeling for patella instability patients. Osteoarthr. Cartil..

[26] Fitzpatrick CK, Baldwin MA, Laz PJ, FitzPatrick DP, Lerner AL, Rullkoetter PJ (2011). Development of a statistical shape model of the patellofemoral joint for investigating relationships between shape and function. J. Biomech..

[27] Dejour H, Walch G, Nove-Josserand L, Guier C (1994). Factors of patellar instability: an anatomic radiographic study. Knee Surg. Sports Traumatol. Arthrosc..

[28] Biedert R, Sigg A, Gal I, Gerber H (2011). 3D representation of the surface topography of normal and dysplastic trochlea using MRI. Knee.

[29] Yamada Y, Toritsuka Y, Yoshikawa H, Sugamoto K, Horibe S, Shino K (2007). Morphological analysis of the femoral trochlea in patients with recurrent dislocation of the patella using three-dimensional computer models. J. Bone Joint Surg. Br..

[30] Hing CB, Shepstone L, Marshall T, Donell ST (2006). A laterally positioned concave trochlear groove prevents patellar dislocation. Clin. Orthop. Relat. Res..

[31] Wise BL (2020). Patterns of change over time in knee bone shape are associated with sex. Clin. Orthop. Relat. Res..

[32] Wise BL (2016). The association of distal femur and proximal tibia shape with sex: the osteoarthritis initiative. Semin. Arthritis Rheum..

