# Supplementary material for: Three-dimensional magnetic resonance imaging-based statistical shape analysis and machine learning-based prediction of patellofemoral instability
Source: Sci Rep. 2024 May 18;14:11390. doi: 10.1038/s41598-024-62143-7 (PMC11102474; doi:10.1038/s41598-024-62143-7)
Supplement: Supplementary file 1 — Supplementary Figure S1. [file 41598_2024_62143_MOESM1_ESM.docx]

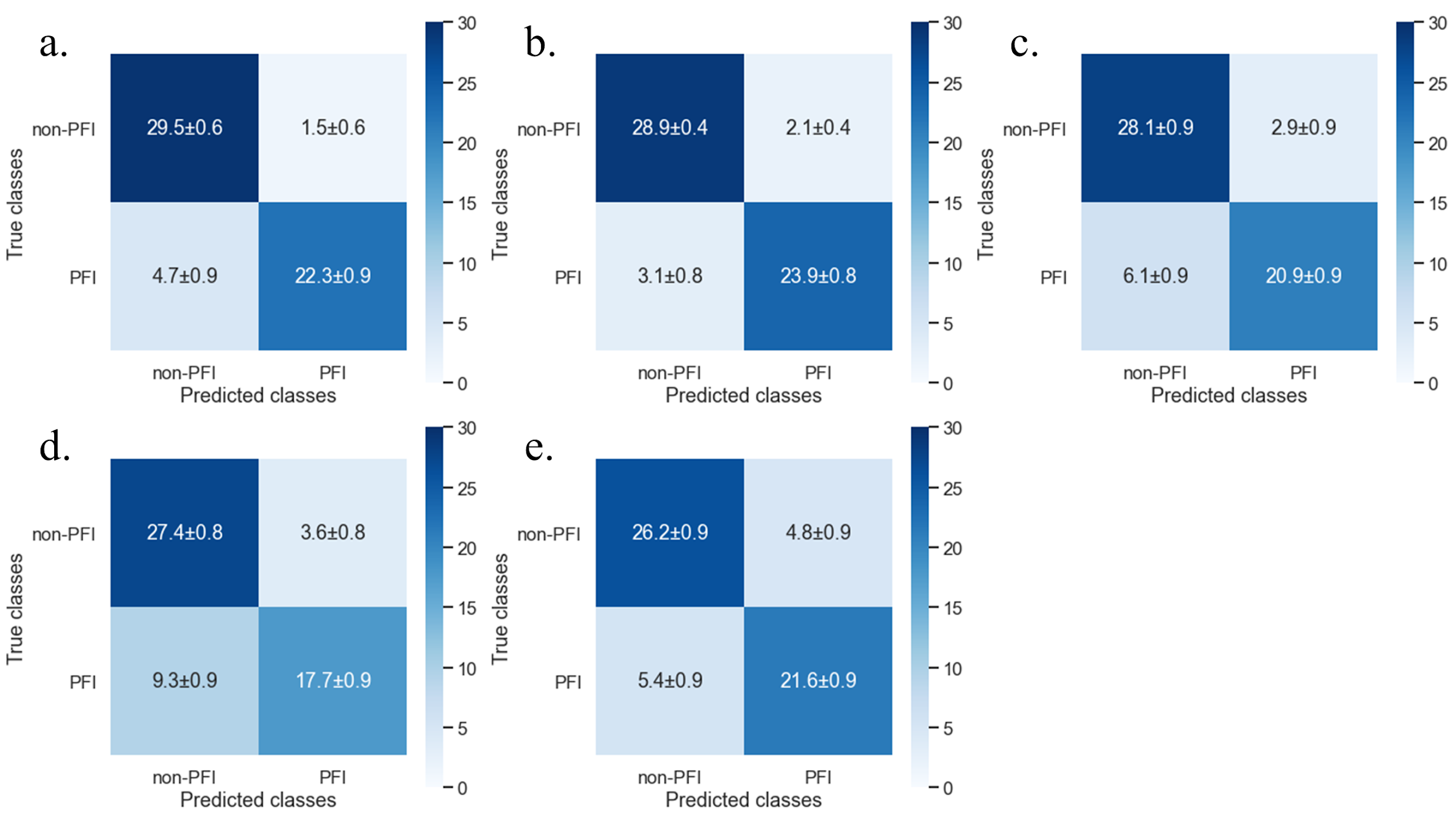


**Supplementary Figure S1.** Confusion matrices show the status of machine learning-based classification for differentiating between patellofemoral instability (PFI) and non-PFI groups using shape components derived from three-dimensional statistical shape analysis, with a linear discriminant analysis (LDA) classifier (**a**), support vector machine (SVM) classifier with a linear kernel (**b**) and rbf kernel (**c**), k-nearest neighborhood (k-NN) classifier (**d**), and random forest (RF) classifier (**e**).
